# A Phosphorescence Quenching-Based Intelligent Dissolved Oxygen Sensor on an Optofluidic Platform

**DOI:** 10.3390/mi12030281

**Published:** 2021-03-08

**Authors:** Fang Wang, Longfei Chen, Jiaomeng Zhu, Xuejia Hu, Yi Yang

**Affiliations:** 1 Key Laboratory of Artificial Micro- and Nano-Structures of Ministry of Education, School of Physics & Technology, Wuhan University, Wuhan 430070, China; wangfang2200@whu.edu.cn (F.W.); coiyhm@whu.edu.cn (L.C.); zhujiaomeng@whu.edu.cn (J.Z.); huxuejiawuda@whu.edu.cn (X.H.); 2 Shenzhen Research Institute of Wuhan University, Shenzhen 518000, China

**Keywords:** optofluidics, dissolved oxygen, phosphorescence quenching, smartphone

## Abstract

Continuous measurement of dissolved oxygen (DO) is essential for water quality monitoring and biomedical applications. Here, a phosphorescence quenching-based intelligent dissolved oxygen sensor on an optofluidic platform for continuous measurement of dissolved oxygen is presented. A high sensitivity dissolved oxygen-sensing membrane was prepared by coating the phosphorescence indicator of platinum(II) meso-tetrakis(pentafluorophenyl)porphyrin (PtTFPP) on the surface of the microfluidic channels composed of polydimethylsiloxane (PDMS) microstructure arrays. Then, oxygen could be determined by its quenching effect on the phosphorescence, according to Stern–Volmer model. The intelligent sensor abandons complicated optical or electrical design and uses a photomultiplier (PMT) counter in cooperation with a mobile phone application program to measure phosphorescence intensity, so as to realize continuous, intelligent and real-time dissolved oxygen analysis. Owing to the combination of the microfluidic-based highly sensitive oxygen sensing membrane with a reliable phosphorescent intensity detection module, the intelligent sensor achieves a low limit of detection (LOD) of 0.01 mg/L, a high sensitivity of 16.9 and a short response time (22 s). Different natural water samples were successfully analyzed using the intelligent sensor, and results demonstrated that the sensor features a high accuracy. The sensor combines the oxygen sensing mechanism with optofluidics and electronics, providing a miniaturized and intelligent detection platform for practical oxygen analysis in different application fields.

## 1. Introduction

Dissolved oxygen is an essential indicator for evaluating water quality, and it is a basis to study the self-purification of water. Rapid, reliable and continuous detection of dissolved oxygen is of great significance in water quality monitoring, aquaculture, industrial production and marine exploitation [1,2,3]. At present, there are many methods for dissolved oxygen detection, among which the most common ones are iodometric titration [4,5], electrochemistry [6] and fluorometry/phosphorometry [7,8,9,10]. The internationally recognized benchmark iodometric method is effective and accurate, but it suffers from large reagent consumption, cumbersome operation and the inability to realize online detection. The most wildly used electrochemical method has advantages of operational simplicity and high sensitivity, but it consumes oxygen, and the electrodes used are susceptible to damage or poisoned and need to be calibrated and maintained regularly, making it difficult to be applied in long-term measurements. Optical oxygen sensors are mainly based on the quenching of fluorescence or phosphorescence by molecular oxygen [11,12,13,14], and phosphorescence-based sensors are much more sensitive than fluorescent sensors by principle [7]. Optical oxygen sensors offer advantages of no oxygen consumption, high detection accuracy, having shorter response time, strong anti-interference ability and convenient to realize sensor miniaturization and are widely applied in the field, including clinical, chemical and environmental monitoring. At present, commercial dissolved oxygen (DO) sensors mainly adopt the fluorescence/phosphorescence quenching-based method, but the measurement is usually carried out manually on the spot. Thus, developing a sensor that automatically and continuously monitor the DO content in water is highly desired.

Platinum(II) meso-tetrakis(pentafluorophenyl)porphyrin (PtTFPP) is one of the ideal phosphorescence dyes that is generally used as the main component of the sensing membrane, because it is resistant to fading, sufficiently photostable and exhibits suitable quenching sensitivity to dissolved oxygen [15,16]. To achieve low limit of detection (LOD), high sensitivity and fast response recovery, the DO sensor’s sensing membrane should exhibit sizeable specific surface, and the polymer substrate of the sensing membrane should have high accessibility of gas molecules [17,18]. Performance of the traditional flat sensing film based on PtTFPP/polydimethylsiloxane (PDMS) is usually unsatisfying, due to the limited surface area. Patterned microstructure-based flexible thin-film sensors have higher potential for practical application, since they have larger specific surface areas, thus enabling higher sensitivity. They can be cost-effective and are more suitable for integration with other components. Therefore, fabricating thin sensing films with a sensor layer composed of ordered microstructures is very attractive [19,20]. Optofluidics is the combination of optics and microfluidics [21,22,23,24,25,26,27,28,29], providing lots of unique advantages for simplifying the micro-electromechanical systems, as well as enhancing their performance [30,31]. Great interest has been drawn to the integration of DO sensors into microfluidic devices. Various patterns of microstructures can be easily introduced into the microfluidic system, thus realizing the integration of the reliable oxygen sensing mechanism into a microfluidic device [32,33], which is then combined with the optical detection to achieve miniaturization of dissolved oxygen monitoring.

In this work, automatic and continuous monitoring of DO contents in water is realized with a phosphorescence-based intelligent sensor on an optofluidic platform. The dye of PtTFPP is used as the oxygen-sensitive phosphor. The DO sensing film with patterned and microstructured arrays is of high sensitivity, as it has a sizeable specific surface area. Instead of using complicated optical or electrical design for phosphorescence detection, the intelligent sensor uses an ultraviolet (UV) light-emitting diode (LED) as the light source to excite the phosphorescence, and a photomultiplier (PMT) counter to collect the phosphorescence signal, combined with two optical filters and an electronic platform to form a reliable optical detection module and realize the automatic and continuous detection of dissolved oxygen. As a part of smart water analysis of our lab [34,35], the sensor utilizes a smartphone APP to serve as the user interface and demonstrate the test results, which makes DO detection more intelligent and convenient.

## 2. Experimental Results

### 2.1. Sensor Setup

**Figure 1:**
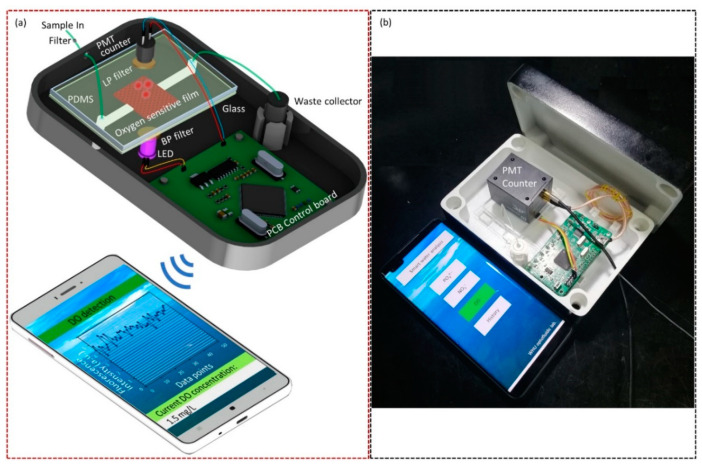
(**a**) Schematic diagram of the fluorescence quenching-based intelligent sensor with patterned microstructures dissolved oxygen (DO) detection. The intelligent sensor is consisted of three functional part: a microfluidic platform, an optical detection module and a smartphone. The mechanism of detection is based on the phosphorescence quenching of the platinum (II) meso-tetrakis(pentafluorophenyl)porphyrin (PtTFPP) sensing film. (**b**) Image depicting the intelligent DO sensor and main interface of the APP on a smartphone.

**Figure 2:**
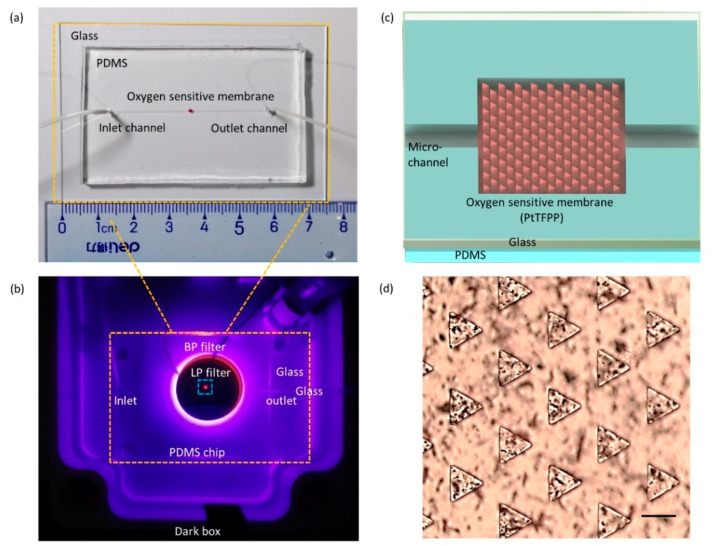
The image of the microfluidic chip incorporated with oxygen-sensitive membrane (**a**) and image shows the optical elements arrangement and phosphorescence emitting of the intelligent sensor (**b**). (**c**) The schematic diagram of the microchannel incorporated with microstructure-assisted PtTFPP/polydimethylsiloxane (PDMS) sensor film. (**d**) The microscope image of patterned microstructures. Scale bar: 50 µm.

As illustrated in Figure 1, this intelligent DO sensor based on patterned microstructures and phosphorescence quenching consists of three major parts: a microfluidic platform, a phosphorescence detection module and a smartphone. The microfluidic platform is composed of an inlet channel, an outlet channel and a flow cell incorporated with an oxygen-sensitive membrane for phosphorescence generation and detection (Figure 2a). The depth of all microchannels is 100 μm. The inlet channel and the outlet channel are 22 mm long and 250 µm wide, and the flow cell with oxygen-sensitive membrane is 1 mm long and 1 mm wide, and many triangular prism microstructures are placed regularly with in the flow cell to enhance the sensor’s efficiency. The microfluidic platform act as both a flow cell for providing a controllable place where the dissolved oxygen interacts with phosphors of PtTFPP immobilized in the PDMS oxygen-sensitive membrane and a phosphorescence detection cell. The optical detection module consists of a high ultraviolet light-emitting diode, a band-wave pass (BP) optical filter, a long-wave pass (LP) filter, a PMT counter and a printed circuit board (PCB). The former four optical elements are arranged vertically and tightly from bottom to top (Figure 1a and Figure 2b). The UV LED (395 nm) is used to excite the PtTFPP phosphors to produce phosphorescence emission. The BP filter (central wavelength: 395 nm) is placed on the top the UV LED to allow only the excited light of the LED to enter the oxygen-sensitive film and minimize the effects of stray light. A PMT counter is used to detect the phosphorescence intensity. An LP filter (590 nm to 1200 nm) is placed on the top of the microfluidic chip and the bottom of the optical window of the photomultiplier, so that only the phosphorescence is emitted into the PMT to reduce the influence of the background light. To achieve miniaturization and intellectualization, the device integrates the microfluidic PDMS chip, a LED, a BP filter, a PMT counter, an LP filter and a PCB control board in a dark box (11 cm × 16 cm × 6 cm) to prevent light interference (Figure 1b). When the sample with different DO content passes through the flow cell incorporated with PtTFPP dye, the oxygen molecules quench the phosphorescence excited by the LED, and the phosphorescence intensity will change. The PMT detect the phosphorescent intensity and convert it into an electric signal. Then, the electric signal is processed by the PCB control board and converted into a digital count, which is finally transmitted through Bluetooth communication and displayed on the smartphone APP.

### 2.2. Principle of Phosphorescence Detection

**Figure A1:**
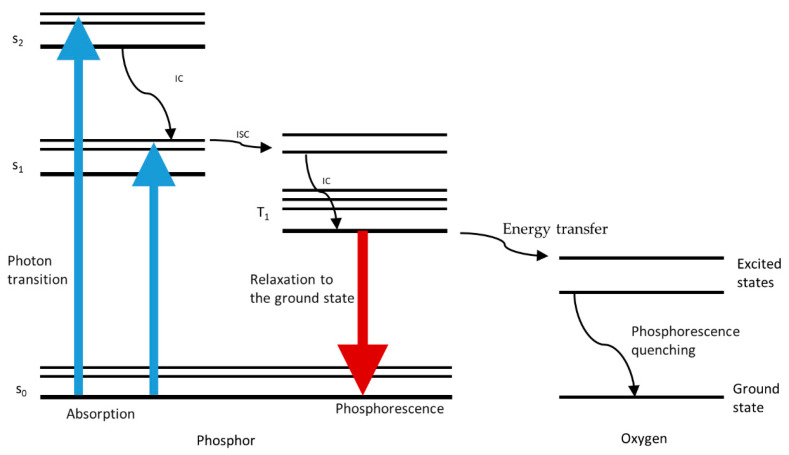
Simplified Jablonski diagram shows phosphorescent generation and main energy transitions during measurement. S_0_: Ground state of the phosphor; S_1_: First excited singlet states of the phosphor; S_2_: Second excited singlet states of the phosphor; T_1_: The excited triplet state of the phosphor; IC: Internal Conversion; ISC: Inter-system Crossing.

The oxygen-sensitive film, which relies on the combination of a phosphorescence dye PtTFPP and a support PDMS polymer layer that is highly oxygen-permeable and chemically stable, is coated on the surface of the microfluidic channel, which is filled with regularly arranged triangular prism microstructures. Figure 2c shows the schematic diagram of the microfluidic platform, which integrated with the oxygen-sensitive membrane. Figure 2d shows the microscope image of the microstructure-based PtTFPP/PDMS sensing film. A total of 145 triangular prismatic microstructures are staggered and orderly distributed in 17 rows and 17 columns in the flow cell with oxygen-sensitive membrane. There is a 110 μm spacing between aligned triangular prisms in each row. The staggered triangular microcolumn arrays are used for enlarging the specific surface area of the sensitive film to enhance its sensitivity. The oxygen-sensitive membrane has high accessibility of gas molecules, so that the oxygen molecules can move freely within the sensing arrays. Figure A1 shows the schematic of the energy transition pathways during the process of oxygen-induced phosphorescence quenching. The phosphors dye of PtTFPP absorbs the excitation light emitted by the UV LED and is transferred from the ground state to higher energy states. The PtTFPP molecule in the single-excited state (S_1_) will transition to its adjacent vibrational energy level T_1_ by means of intersystem conversion (I_SC_). In the absence of oxygen, a molecule in the T_1_ energy level will likewise return to the ground state by radiative transition, emitting light with a central wavelength of about 645 nm [15,16,17]. With the existence of quencher oxygen, the energy is transferred from the phosphors dye of PtTFPP to the oxygen, and, thus, no phosphorescence is emitted out. As a result, the intensity of emitted phosphorescence decreases with the increase in DO concentrations. The oxygen-quenching process occurred in the flow cell incorporated with oxygen-sensitive film could be described by the Stern–Volmer formula:

(1) $$I_{0}/I=1+K_{\mathrm{SV}}*C\left(O_{2}\right)$$

where I stands for the phosphorescence intensity, I_0_ stands for the reference phosphorescence intensity in oxygen-free water, K_SV_ stands for the Stern–Volmer constant and C (O_2_) stands for the DO concentration in solution. Meanwhile, the Stern–Volmer plot, which means a plot of the ratio of unquenched and quenched luminescence intensity (I_0_/I) versus oxygen concentration C (O_2_), will be linear with an intercept at 1 and a slope of K_SV_.

**Figure 3:**
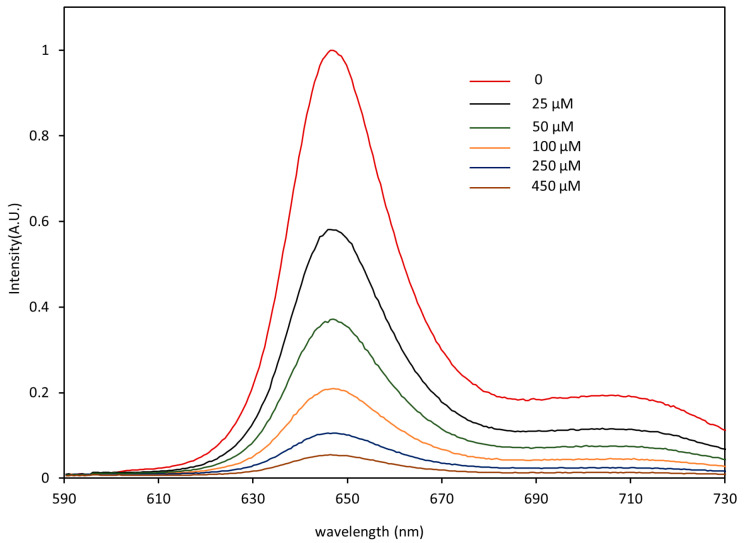
Emission spectra of the PtTFPP-based sensing film with patterned microstructures in waters with different DO concentrations.

Figure 3 shows the phosphorescence emission spectra of the PtTFPP-based oxygen-sensitive membrane with patterned microstructures, which illustrates the remarkable light intensity-changing features in waters with different DO concentrations and high phosphorescence brightness in low DO environment. Water samples with different DO concentrations (0, 25 μM, 50 μM, 100 μM, 250 μM, 450 μM) were prepared through the aeration process by ultrapure nitrogen and oxygen based on Henry’s law; the detailed preparation process was reported in our previous work [36]. 

### 2.3. Sensor Calibration

**Figure 4:**
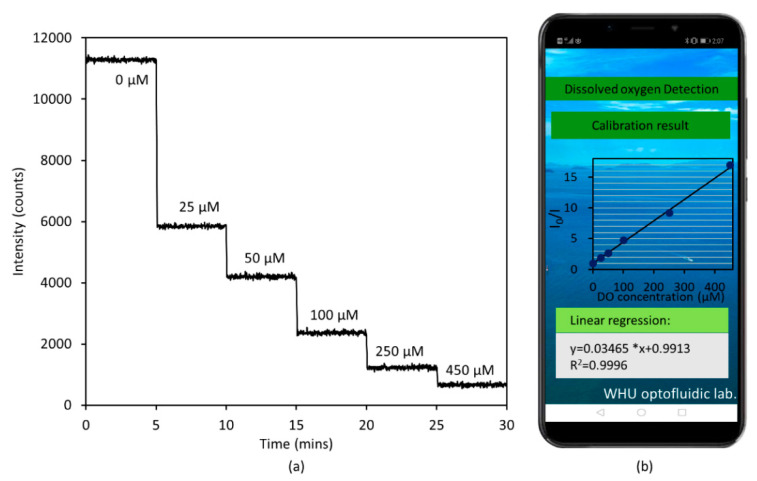
(**a**) Response of the intelligent DO sensor to different oxygen concentrations. (**b**) The Stern–Volmer plot of the intelligent sensor.

The first step of DO measurement is sensor calibration, which means obtaining the Stern–Volmer plot. After completing the calibration settings on the mobile APP, water samples with different DO contents were passed through successively to initiate the calibration. Each water sample was continuously injected for 5 min to allow the DO to fully immerse into the PtTFPP membrane. The flow rate was 100 μL/min. The phosphorescence intensities were recorded once every second. Figure 4a shows the response of the intelligent DO sensor measured at room temperature (22 °C). It was observed that the phosphorescence intensity has a remarkable dependence on the concentration of DO. The Stern–Volmer plot shown in Figure 4b has a linearity of 0.9996 and a Ksv = 34.65 L/mmol, indicating that the intelligent DO sensor exhibits a good linear correlation in DO range from 0 to 450 μM. The response sensitivity in DO measurement is given by I_0_/I_s_, where I_0_ represents the sensor responses in oxygen-free water (0) and Is represents the sensor responses in oxygen-saturated water (450 μM). Here, the sensitivity for the presented intelligent sensor is 16.9. The LOD of the sensor is governed by both the Stern–Volmer constant (K_SV_) and the resolution of the intelligent sensor. The uncertainty in light intensity measurement of the intelligent sensor was 0.43%, and then the LOD of the sensor could be calculated as 0.013/K_SV_, resulting in a LOD of 0.01 mg/L (0.37 μM). It could be seen that the intelligent DO sensor exhibits a higher sensitivity at a lower DO concentration. Thus, it can achieve higher accuracy when applied in an environment with low dissolved oxygen concentrations such as sewage. 

### 2.4. Sensor Response Time and Performance

**Figure 5:**
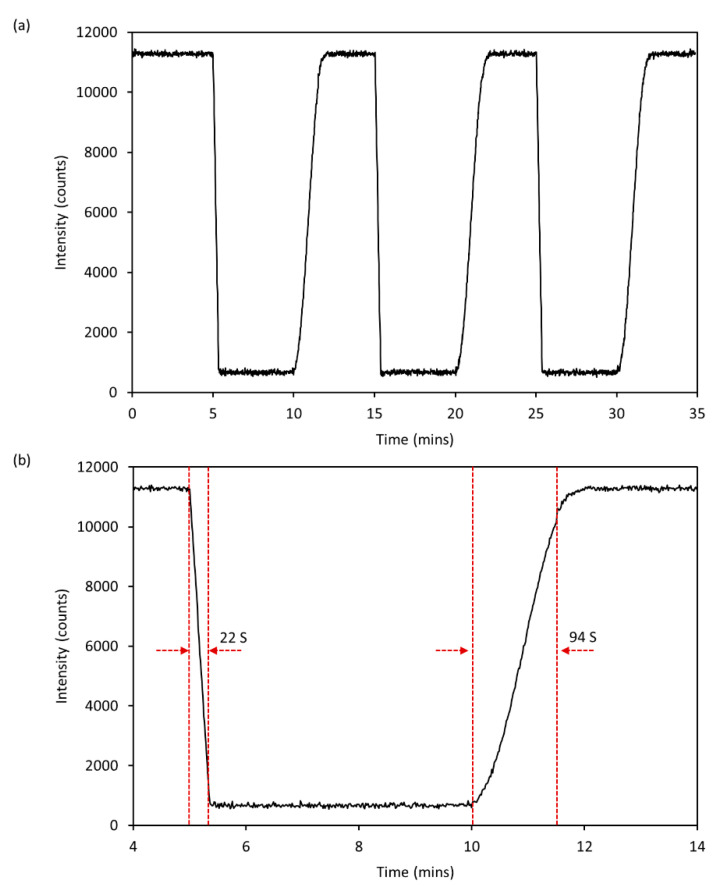
(**a**) Reversible response of the intelligent DO sensor to O_2_-free and O_2_-saturated water samples. (**b**) The partial enlarged curve of (**a**).

A reversible and fast response of the intelligent DO sensor to the change of oxygen concentration was found and is shown in Figure 5. The reversible response of the intelligent DO sensor was investigated by the alternative injection of oxygen-free and oxygen-saturated water samples. Phosphorescence intensity was recorded once per second. Results demonstrated the good reproducibility of the intelligent DO sensor. After three measurement cycles, the phosphorescence intensity of the DO sensor in oxygen-free and oxygen-saturated water shows no decrease. The intelligent DO sensor takes about 22 s and 93 s, respectively, to accomplish 90% of the overall phosphorescence intensity variation from oxygen-free to oxygen-saturated water samples, and vice versa. It could be concluded that the quenching time of oxygen on the indicator is shorter than the recovery time of the indicator. The main factor affecting the DO sensor’s response time is the diffusion rate of oxygen in the sensing membrane. Therefore, the shorter response time of the DO sensor than its recovery time may be due to the fact that the diffusion time of oxygen is much shorter than that of other gases [37].

**Figure 6:**
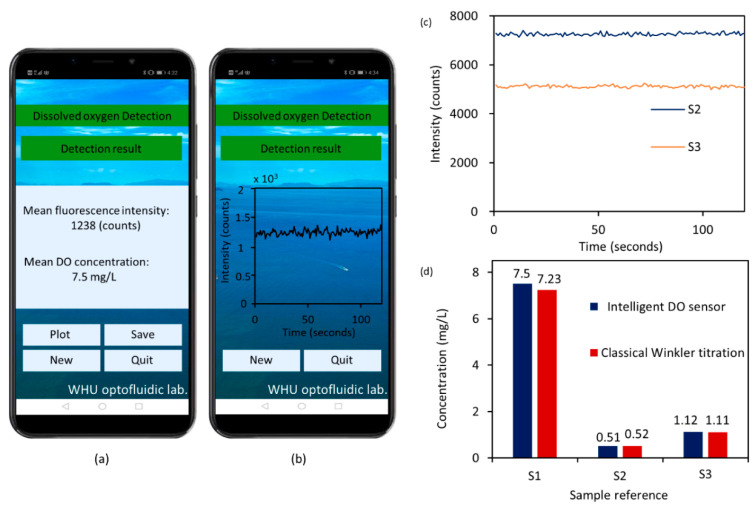
(**a**) The APP interface for DO detection result of the lake water sample (S1). (**b**) The APP interface shows the plot of the DO detection result for S1. (**c**) Phosphorescent intensity for S2 and S3; the data was derived from the mobile APP. (**d**) Comparison of the proposed method and reference method.

The DO contents in a lake water sample (S1) and two sewage water samples (S2 and S3) were analyzed using both the intelligent sensor and the classical Winkler titration method. Figure 6 shows the results. Noticeably, before each test, the chip was rapidly flushed (100 μL/min) with the analyte for 3 min to allow the DO to be in full contact with the sensitive film and to help expel bubbles and thus reduce the impact of bubbles on phosphorescence intensity detection. Each sample was measured 120 times in 2 min to reduce the random error and improve measurement accuracy. Figure 6a,b show the APP interface for measurement results of S1. Figure 6c shows the detection results of S2 and S3. The phosphorescent intensity data was derived from the mobile APP. It was observed that the DO contents obtained by the intelligent sensor fit well with the results obtained by the classical Winkler titration method (Figure 6d), and the measurement errors for all determination results was in the range from 0.9% to 3.7%, indicating the high accuracy of the intelligent DO sensor.

## 3. Materials and Methods

### 3.1. Chemicals

Milli-Q water (18.25 MΩ·cm, DI water) was used to prepare all solutions. The porphyrin dye PtTFPP was purchased from Frontier Scientific Inc. (Logan, UT, USA). Three natural water samples were used to demonstrate the performance of the intelligent sensor in monitoring DO contents of actual water. Sample 1 was sampled from the East Lake of Wuhan in January; Samples 2 and 3 came from local sources. Natural water samples are filtered by a 0.45 μm pore size filter before detection. All of the natural water samples were sealed hermetically before use to minimize the impact on detection accuracy. 

### 3.2. Oxygen-Sensitive Membrane and Microfluidic Chip Fabrications

The PtTFPP dye was first completely dissolved in toluene and then mixed with uncured PDMS prepolymer (A: B = 10: 1, Sylgard 184, Dow Corning, Midland, Michigan, USA). The content of PtTFPP was 1mg/g in the PDMS. Next, the mixture was stirred for half an hour with a magnetic stirrer. A small amount of the above mixture was dropped onto the oxygen-sensitive membrane cell of the precarved silicon mold, fabricated using a standard soft lithography technique [17,36], and then placed in a vacuum oven for degasification. Subsequently, a support layer of the pure mixture of uncured PDMS prepolymer was poured on the top of the above PtTFPP-based oxygen-sensitive membrane. Then, the fabricated PDMS chip containing PtTFPP film was stored in an oven at 75 °C for 30 min to enhance the bonding. The microfluidic chip was ready to use after being inserted with the fluidic tubings. 

### 3.3. Instruments

**Figure A2:**
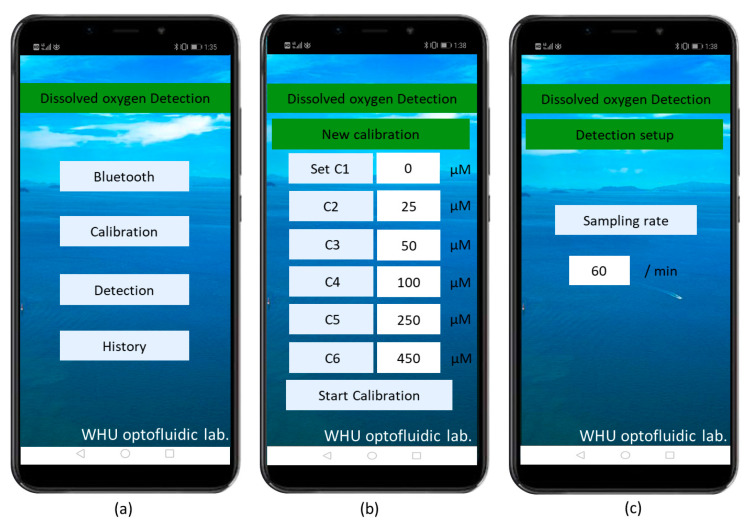
The images show each functional interface of a smartphone APP. (**a**) The main interface of the APP. (**b**) The interface for calibration setup. (**c**) The interface for detection setup.

The intelligent dissolved oxygen sensor was controlled by a custom-designed and double-layered electronic circuit board based on a microcontroller (STM32F, STMicroelectronics). An ultraviolet LED (395 nm, LEUVA35T01UL00, LG, Seoul, Korea), a band-pass filter (BP395-20 nm, Rayan, Changchun, China), a PMT photon counter (JPC-1050-TEC, Joinbon, Wuhan, China) and a long-wave pass filter (LPF590 nm, Rayan, Changchun, China) were used for phosphorescence intensity detection. It should be noted that the center of the two filters, the LED and the optical window of the PMT should be accurately aligned to reduce the impact of stray light, and the PMT should be fixed on the periphery of the long-pass filter using optical glue. An image was obtained by the inverted microscope (Ti-E, Nikon, Tokyo, Japan) to illustrate the triangular prism microstructure in the microfluidic chip. A spectrograph system consists of a Charge Coupled Device (CCD) camera (Newton 920, Andor Tec., Belfast, UK) and an Andor’s line of Shamrock imaging spectrographs (Shamrock 303i, Andor Tec., Belfast, UK) was used to record the phosphorescence emission spectra of the PtTFPP oxygen-sensitive membrane with patterned microstructures. Water samples were pumped into the microfluidic chip at accurately controlled flow rates with a portable syringe pump (LD-P2020II, Lande, Shanghai, China). An android phone was used as the intelligent control terminal. Figure A2 shows each functional interface. 

## 4. Discussions

Table 1 shows the performance of dissolved oxygen sensors using different detection mechanisms. Compared with other DO sensors, the intelligent DO sensor presented here is more portable, because it abandons the use of bulk laboratory equipment for data processing and a special customized display panels as the user interface. In addition, it features a low detection limit (0.01 mg/L), rapid response (22 s) and high sensitivity (16.9) in low dissolved oxygen concentration environment, making it especially suitable for monitoring dissolved oxygen in sewage, aquaculture and biological detection. The presented intelligent DO sensor adopts the phosphorescence intensity-based measurement, and the method is wildly used due to its simplicity and low cost. However, it has major disadvantages, including susceptibility to light source and detector drift and degradation or leaching of the dye. The measurement error caused by fluctuations of optical signal can be effectively decreased by calibrating before measurement and averaging multiple measurements [38,39]. Moreover, it has been confirmed that the phosphor of PtTFPP used here has perfect photochemical stability [40,41], and PtTFPP/PDMS sensor film is extremely stable at room temperature [17]. 

All experiments were conducted at room temperature. A further systematic investigation is necessary to explain the effects of interferences such as temperature, salinity, pH and bubbles on the performance of intelligent sensors, and this research is being conducted in our laboratory. In addition, the integration of DO sensors with temperature, salinity and pH sensors to realize automatic and real-time dynamic compensation and correction of temperature, salinity, pH and other interference factors is the development trend of intelligent dissolved oxygen sensors [11], which is also the focus of our future research. 

## 5. Conclusions

In conclusion, we demonstrated a phosphorescence quenching-based intelligent sensor on an optofluidic platform for continuous detection of dissolved oxygen in water. The miniaturization of DO monitoring was achieved by combining the DO phosphorescence sensitive film of PtTFPP with optofluidics and electronics. The intelligentization was then realized by the utilization of a smartphone. The Stern–Volmer response of the sensor and its performance in measuring DO content in natural water samples were investigated. Results show that the sensor exhibits excellent sensitivity, due to the coating of a DO-sensing film with indicator PtTFPP on the microchannel, composed of patterned PDMS microstructure arrays, and the combination with a reliable phosphorescence detection module. The intelligent sensor has a detection range of 0 to 14 mg/L, a low LOD of 0.01 mg/L and a quick response (22 s). For the detection of natural water samples, the intelligent sensor achieves similar detection results with the classical Winkler titration method, but the former is simple in design, demands less reagent and has higher integration and intelligence. Therefore, the phosphorescence quenching-based intelligent DO sensor on an optofluidic platform has a good application prospect in water quality and biomedical analysis.

## Figures and Tables

**Table 1:** Comparison of the intelligent DO sensor with other DO detection techniques.

Dissolved Oxygen Detection	Electrochemical Sensor [42]	Optofluidic Sensor [36]	Optical Sensor [9]	Commercial Sensor [11]	Intelligent Senor
Principle	Electrode polarography	Colorimetry	Phosphorescence-quenching	Fluorescence-quenching	Phosphorescence-quenching
Sensitivity	64.6 nA·L·mg^−1^	7.5 nm·L·mg^−1^	I_0_/I > 4.6	-	I_0_/I = 16.9
Detection range	0.3 to 8.4 mg L^−1^	0 to 16 mg L^−1^	0 to 11.6 mg L^−1^	0 to 20 mg L^−1^	0 to 14.4 mg L^−1^
Detection Limit	0.2 mg L^−1^	3.52 ug·L^−1^	0.03 mg L^−1^	0.01 mg L^−1^	0.01 mg L^−1^
Response time	7.5 s	-	< 1min	40 s	22 s
User Interface	-	-	PC	Customized display panel	Smartphone APP

## Data Availability

The data presented in this study are available on request from the corresponding author. The data are not yet publicly available as preliminary examples to establish proof-of-concept.
